# Aging and the prevalence of ‘ironic’ action errors under avoidant instruction

**DOI:** 10.1371/journal.pone.0213340

**Published:** 2019-03-21

**Authors:** Lauren M. Potter, Madeleine A. Grealy

**Affiliations:** 1 Department of Psychology, Heriot-Watt University, Edinburgh, Scotland, United Kingdom; 2 School of Psychological Sciences and Health, University of Strathclyde, Glasgow, Scotland, United Kingdom; Cornell University, UNITED STATES

## Abstract

Action errors can put older adults at risk of injury. Our study is the first to investigate whether older adults are more prone than younger adults to making ‘ironic’ motor errors (i.e., actions they have been instructed *not* to perform), or over-compensatory motor errors (e.g., moving more to the right when instructed not to move to the left). We also investigated whether error patterns change under cognitive load, and assessed whether age effects in the ability to inhibit a prohibited action are comparable to the age decrements found in the ability to inhibit a natural perception-action coupling in the Simon task. Sixty-four older (Mean = 70.64 years, SD = 5.81) and 39 younger (Mean = 28.74 years, SD = 16.39) adults completed an avoidant instruction line-drawing task (with and without cognitive load), and the Simon task. Older adults showed significantly slower inhibition times than younger adults on the Simon task, as expected, and in line with previous research. Surprisingly, however, older adults outperformed younger adults on the avoidant instruction task, producing fewer ironic and over-compensatory errors, and they performed similarly to the younger adults under cognitive load. Age-related decrements on the Simon but not the avoidant instruction task suggests that the two different types of motor tasks involve different subtypes of inhibition which likely recruit independent cognitive processes and neural circuitry in older age. It is speculated that the older adults’ superior ability to inhibit a prohibited action could be the result of age-related changes in distractibility.

## Introduction

As we age we experience declines in cognitive [1] and motor [2] abilities but relatively little is known about how these changes interact to affect the performance on daily activities. Cognitive resources are fundamental to all levels of action control, from perceiving [3–4] and planning [5–6] even basic actions to generating and maintaining normal and consistent movement patterns [7], and whilst the literatures on cognitive and motor ageing have expanded rapidly they have done so largely independently leaving gaps in our knowledge about the interplay between the two. This is particularly true for the inhibitory processes that are fundamental to both cognitive functioning [8] and goal directed action [9].

This study focuses on cognitively-driven motor errors that have previously been described in younger adults. These errors arise from a failure to inhibit instructions to avoid a particular movement and we investigate, for the first time, whether these errors become more prevalent with age and whether this type of inhibition is related to the ability to inhibit an automatic motor response.

Instructing someone *not* to perform a particular action can, ironically, make them more likely to execute that action, particularly if they are under cognitive load. These ‘ironic’ action errors have been demonstrated extensively in younger adults but have yet to be examined in older adults. When runners are told not to leave the starting blocks early, they are more likely to trigger a false start [10]; when told not to miss a penalty kick, footballers will miss more [11–13]; when told not to wobble, dancers will wobble more [14]; when told not to miss in a specified location (“down the T” or “out wide”), tennis and darts players miss more often in those locations [15–16]; and when told not to over-shoot, golfers and hockey players will over-shoot more [16–19]. According to the Ironic Processing Model [20], this phenomenon occurs as a result of a competition between two interacting cognitive processes which direct our attention and subsequent actions; an effortful intentional operating process that consciously directs our attention towards desired thoughts and goals, and a monitoring process that subconsciously attends to the possible thoughts and actions which could cause us to fail. When we are told not to make an action the operating system searches for thoughts about actions to replace the prohibited one; that is we consciously try and focus on anything but the unwanted action. At the same time the monitoring process automatically searches for ways to fail and checks our consciousness to make sure we are suppressing thoughts about the prohibited action. Unfortunately, this monitoring process can also influence the accessibility of conscious thoughts and increase our sensitivity to the very thought we are trying to inhibit. So the more effort we put into trying to suppress the thought of the prohibited action, the more active the monitoring system becomes until thoughts about making the prohibited action become conscious and it is executed.

If ironic errors are a form of inhibitory failure then it is possible that their prevalence will increase with age as ageing has been shown to effect both cognitive and motor tasks that require inhibitory control. Age-related changes in the ability to inhibit dominant motor responses have been demonstrated using stop signal paradigms [21–28] and the Simon task [29], which assesses the ability to inhibit a natural perception-action coupling. Typically in the Simon task participants are required to inhibit the natural tendency to make a motor response based on the location of a cue and respond instead to the stimulus colour. Findings show that reaction times are shorter when the stimulus and response are spatially compatible (i.e., a left side response is required for a stimulus displayed on the left) compared to when they are incompatible (i.e., a right side response is required for a stimulus displayed on the left). Older adults find it more difficult than younger adults to inhibit the faster spatial response in favour of the slower colour response, even after accounting for age-related slowing of information processing speed [30–34]. Age-related decrements in the ability to inhibit planned reaching actions [35] and copied manual actions [36] have also been reported and increases in the magnitude of motor inhibition increases with age [36].

Age differences on cognitive inhibitory tasks have also been investigated, but the pattern of findings is less clear. For example, studies using directed forgetting tasks show that older adults are less effective than younger adults at inhibiting information that was cued to be forgotten [37]. Similarly, the increased familiarity from repeatedly reading a word leads older people to make errors in recollecting whether they had read or heard the word previously [38]. On more complex forgetting tasks, greater age decrements are found on encoding compared to retrieval tasks [37], further supporting differential age effects in inhibition. In contrast, older adults report fewer instances of having to inhibit unwanted thoughts than younger adults, and the frequency and duration of these remain stable over time [39]. No age-related differences are found however in the ability to inhibit thinking about a previously learned word association [40]. Similarly, whilst older adults report using thought inhibition less frequently than younger adults, these age effects are explained by trait anxiety and rumination [41].

The notion of a single global age-related inhibitory deficit [42] does not seem to fit with the findings across the cognitive and motor domains, and comparisons between different inhibition subtypes have revealed age affects for some but not other types. For example, negative priming studies [43] show that the inhibition of target location is preserved with older age, whereas the inhibition of target identity is not. Similarly, in a comparison across cognitive tests, older adults were slower to inhibit planned key-press responses in stop-signal tasks, and produced more perseverative errors on the Wisconsin Card Sorting Test, but performed comparably to younger adults on Stroop and negative priming tasks [27]. Thus, different subtypes of inhibition may be differentially affected by age, and recruit different underlying neural mechanisms. This is supported by research [43] suggesting that age-related declines in the inhibition of target identity, but not target location, may be due to greater age-related degeneration within the ventral occipito-temporal neural pathway, relative to the dorsal occipito-parietal pathway.

A second type of action error can arise from avoidant instructions and is known as an over-compensatory error. This was demonstrated using a simple line-drawing task where young participants traced an invisible straight line between two dots [44]. When instructed to move straight and avoid moving to the left or right of the invisible line 25% of participants made ironic errors (i.e., when instructed not to go left they moved more to the left), but 65% made over-compensatory errors (i.e., when instructed not to move to the left they moved more to the right). Giving the participants an additional cognitive task changed the movement patterns in over half of the participants with 33% changing from making errors under no load to being accurate under cognitive load, suggesting that distraction from the avoidant instructions aided performance. Interestingly, under both conditions the majority of participants were unaware that their movement patterns changed when they heard the avoidant instructions, suggesting this was implicitly rather than consciously controlled.

Whilst the Ironic Processing Model [20] can account for the ironic errors it does not explain this greater prevalence of over-compensatory compared to ironic errors. Instead, these can be explained by the implicit compensation hypothesis [45], which argues that over-compensatory errors are caused by negatively worded instructions exaggerating the importance of making an action error, leading to the misconception that it is less risky to err away from the forbidden direction rather than towards it. This ‘play safe’ over-compensation strategy may be a more likely default control of action than the ironic model’s implicit monitoring system. With increasing age, and an awareness of poorer inhibitory control this strategy may be one that is adopted by older adults. As the prevalence of action errors induced by avoidant instruction in older age is not yet known our first aim was to investigate whether the tendency to make ironic and over-compensatory motor errors under avoidant instruction changes with age. Two competing predictions were tested; in the first it was reasoned that age-related declines in working memory capacity would limit the resources available for controlled operating processes leaving the monitoring process to assume control of action under avoidant instruction. Therefore, it was predicted that older participants would make significantly more ironic errors than younger adults. The second prediction was that in order to compensate for changes in their inhibitory abilities older adults would more readily adopt a ‘play safe’ strategy and produce more over-compensatory errors than younger adults. In both cases it was also predicted that the magnitude of the errors made by the older adults (the distance they deviated away from the line on the line-drawing task [44]), would be greater than that shown by younger adults.

Our second aim was to investigate whether there are age differences in the ability to inhibit avoidant instructions under increased cognitive load. In line with previous research [44] we did not expect to see a discernible trend across the low and high cognitive load conditions for the younger adults, however, for the older adults additional cognitive demands should place extra pressure on the resources available for controlled operating processes. If the Ironic Processing Model accounts for their behaviour then older adults should make more ironic errors under higher cognitive load than no load. Similarly, if the older participants were adopting a ‘play safe’ strategy then we could expect an increase in the number and/or the extent of over-compensatory motor errors under higher load. Alternatively, if the secondary task provided a distraction from the avoidant instructions then fewer errors under cognitive load would be expected.

Our third aim was to assess whether inhibiting avoidant instructions and inhibiting a spatially driven motor response are related. The Simon task requires the effortful inhibition of a spatial response. Participants are instructed to respond to the colour of stimuli rather than location but in completing this task it is possible that they use self-generated avoidant instructions (‘don’t press the button on the same side’) to try and avoid errors. This could evoke the monitoring system similarly to the avoidant instruction task. If this is the case then age-related declines seen on the Simon task could, in part, be explained by a failure to inhibit self-generated avoidant instructions. As an initial step to assess this we predicted that if the two tasks are related then inhibition time measured on the Simon task would be significantly correlated with the prevalence, and potentially magnitude, of ironic errors under both load conditions.

## Methods

### Participants

We recruited 130 participants (complete data sets were collected from 103) through various organisations in central Scotland. The mean age was 70.64 years (*SD* = 5.81) for the older (*N* = 64; 30 males, 34 females), and 28.74 years (*SD* = 16.39) for the younger sample (*N* = 39; 17 males, 22 females). The exclusion criteria were a Mini-Mental State Examination [46] score of 27 or less, movement disorders or upper limb mobility restrictions, uncorrected eyesight, and medications likely to affect performance. Local ethical approval was granted from the Ethics Committee at The University of Strathclyde. Participation was voluntary. All participants gave written informed consent.

### Procedure

Participants completed both the line-drawing task and Simon task in a randomized order. Developed in a previous study [44], the line-drawing task assessed participants’ ability to inhibit thoughts about making a movement error. A circle 22cm in diameter, with two opposing dots positioned to indicate the end points of an invisible line running vertically through the centre of the circle, was displayed on a computer monitor. We told participants to imagine an invisible straight line connecting the two dots, then use a mouse to move a cursor back and forth between them, tracing the invisible line as accurately as possible. We asked them to make movements at a pace of approximately one move per second and to move in a steady, fluent rhythm, landing the mouse cursor on the dot before reversing. We told them the computer programme was recording their movements and they would receive additional instructions to avoid making specific movements prior to some trials. It was emphasised that obeying these avoidant instructions was of secondary importance to their goal of tracing the line accurately and that avoidant instructions were not feedback for performance on previous trials. Participants completed three blocks of trials; one control and two with avoidant instructions. At the start of the control block they were instructed to “make sure you move in a straight line. Ready, steady, go”. Participants then moved the mouse between the two dots for six seconds, making approximately five or six full movements. They were then told to stop. This was repeated a further five times so each participant made approximately 30–36 control movements. Similarly for the two avoidant conditions the participants were told “make sure you do not move to the left of the line” or “make sure you do not move to the right of the line” prior to each 6 second trial. The order in which the blocks were completed was randomized. Participants made approximately 60–72 movements in total under avoidant instructions.

Each participant completed the whole task twice in counterbalanced order, once with the instructions as detailed above (low load condition) and once when they were also asked to remember a 7-digit number (high load condition). This number was presented for seven seconds prior to the start of the trial and they were told to rehearse it whilst completing the line-drawing task and that they would be asked to recall it at the end. During each trial the x and y coordinates of the mouse were captured using Labview software with a sampling frequency of 200Hz. Following data collection a Labview analysis programme calculated the leftward and rightward cursor deviations from the target line (cm) during each movement, as well as movement amplitude, duration, peak velocity, and end-point positions.

The Simon task [29] was used to measure perceputo-motor response speed and the ability to inhibit a perceptually driven motor response. Participants were informed that a red or green circle would appear on either the left or right side of the computer screen and that they should press the corresponding colour coded key on a Cedrus RB-730 response pad as quickly as possible. The stimuli and response were either congruent (e.g., a green circle presented on the left of the screen required the left green response button to be pressed) or incongruent (e.g., a green circle on the right of the screen required the left green response button to be pressed). At the start of each trial a fixation cross appeared in the centre of the screen and after a delay of unpredictable length this disappeared and either the red or green circle appeared to the left or right and remained on the screen until the participant pressed a response button. Participants responded using their two index fingers. The positions of the response buttons were randomized so that the red button was on the right for half of the participants and on the left for the other half. Participants were given a short practice session of five congruent trials, followed by 145 trials comprising 95 congruent and 50 incongruent trials presented in a randomized order.

### Data processing procedures

#### Avoidant instruction line-drawing task

**Fig 1:**
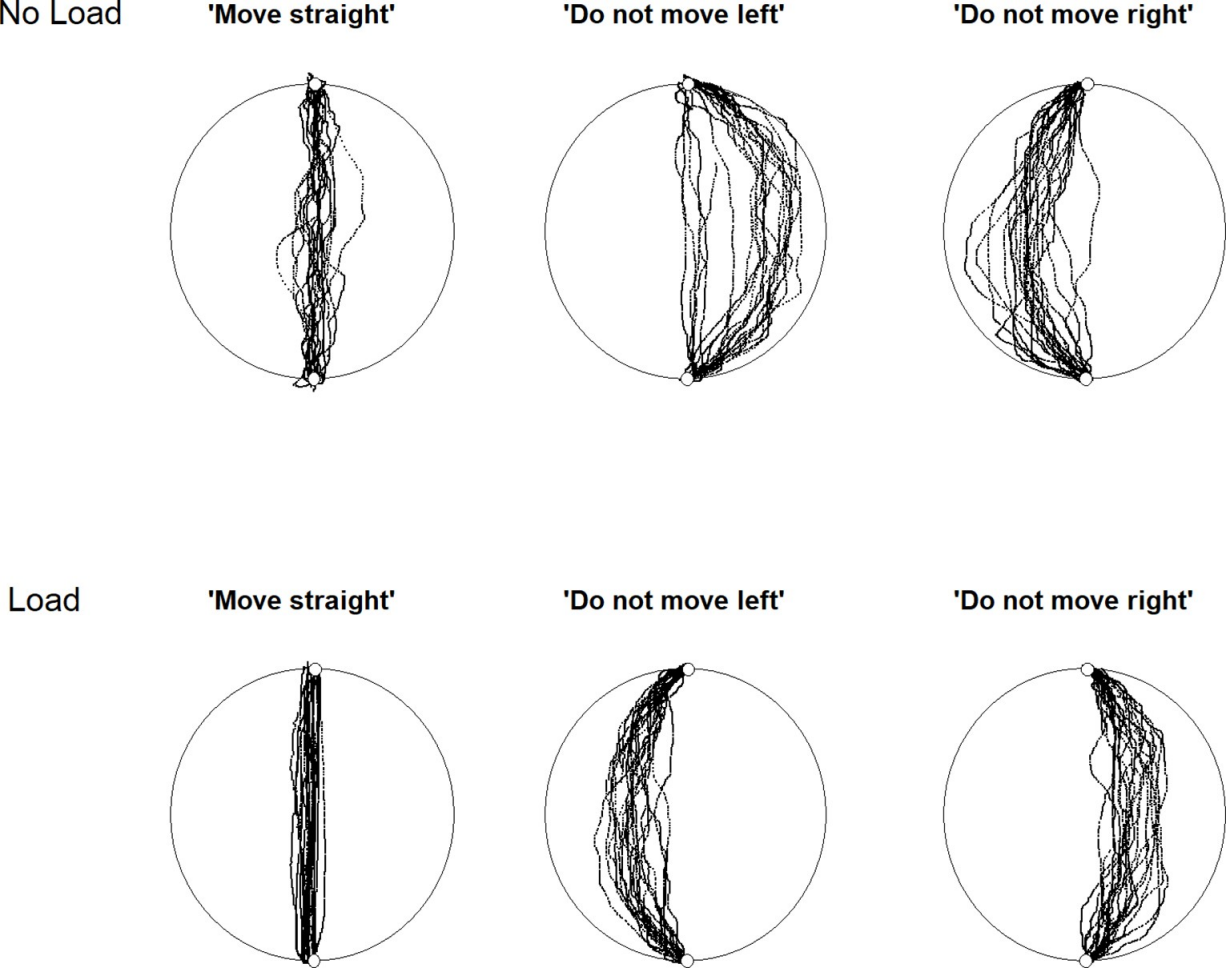
An illustration of the movements made by one older participant. Under the no load condition they made over-compensatory errors and under load they made ironic errors.

Fig 1 provides an example of the data collected from one older participant. Three sets of measures were derived from the data for each participant; the percentage of movements with ironic or over-compensatory errors, magnitude of movement errors, personal performance category.

Incomplete movements were removed. Complete movements consisted of approximately 200 data points. On each movement every x value of the cursor was subtracted from the x value for the target midline. This give a series of leftward and rightward deviations relative to the central line for each movement. These leftward and rightward deviations were then summed to give a total leftward and total rightward deviation score for each movement. To establish whether total deviations scores in the avoidant instruction conditions were greater or less than those in the control condition, we first calculated the average leftward and rightward summed deviations made over all the control movements and under each load condition. Then for each movement made under avoidant instruction, the mean control summed deviations were subtracted from the summed leftward and rightward deviation scores. This allowed us to calculate the magnitude of the ironic errors (leftward deviations on trials with instructions ‘avoid moving left’ and rightward deviations on trials with instructions ‘avoid moving right’) and compensatory errors (vice versa) for each movement. Average magnitudes for ironic errors and compensatory errors under no load and load were then calculated for each participant and then each age group.

To assess the percentage of ironic and over-compensatory errors, each movement made under avoidant instruction was categorized as being ironic, over-compensatory, or accurate. If both summed leftward or rightward deviations were within +5% of the control values then the movement was classified as accurate. If the magnitude of ironic error was greater than +5% of the control and also greater than the over-compensatory error then the movement was categorized as ironic, and vice versa for the categorization of over-compensatory errors. If both the leftward and rightward deviations were greater than +5% of the control values the trial was discarded. It was decided to exclude participants if more than three trials were discarded on this basis, but this did not occur. Once all movements were categorized, percentages of ironic, over-compensatory, and accurate movements were calculated for each individual.

Finally, to categorise the overall performance of each person in each condition we used four categories; ironic, over-compensatory, accurate or random. The criteria adopted was based on previous research [44]. We ruled that if the participant had more than 45% of their movements as one type and no more than 40% of movements in either of the other two types, then their performance was classified as the dominant error type. For example, if a person had 54.37% of their movements with ironic errors, 27.27% of movements with over-compensatory errors and 18.36% accurate movements, then they were classified as an ironic performer. This system was used to categorise ironic, over-compensatory and accurate performances, and for those participants where there was no predominant pattern (for example, ironic = 29.08%, over-compensatory = 31.21% and accurate = 39.71%) their performance was classified as random.

A series of data checks ruled out any age, sex, or condition related differences in movement amplitudes, movement durations, peak velocities and end-point positions. Further checks ruled out any tendencies to move in one direction regardless of the instruction. There were no significant differences in the deviations made when instructed ‘don’t move to the left’ compared to ‘don’t move to the right’, therefore data were pooled across instruction type and deviation direction; left deviations with ‘not left’ instruction were averaged with right deviations with ‘not right’ instruction to give an ironic deviation score, and right deviations with ‘not left’ instruction were averaged with left deviations with ‘not right’ instruction to give an over-compensatory deviation score. There were also no significant age differences in digit recall. An additional check assessed whether switching block influenced performance; statistical analyses were conducted with all data, then repeated but with the exclusion of the first trial from each block of six. This did not change the pattern of significant findings thus all data are reported here.

#### The Simon task

Trials on which participants made an error (i.e., pressed the green button in response to the red stimuli or no button was pressed) were removed. Outliers (± 2 SD) were also removed and an average response time and inhibition time (RT incongruent–RT congruent) was calculated. Inhibition time was then expressed as a proportion of RT congruent time to account for general slowing in older age.

## Results

### Avoidant instruction errors

The initial set of analyses were conducted to test two competing predictions; first was that as age-related declines in working memory capacity would limit the resources available for controlled operating processes older participants would make significantly more ironic errors than younger adults under no load. The second was that older adults would be more likely to adopt a ‘play safe’ strategy and produce more over-compensatory errors than younger adults.

**Fig 2:**
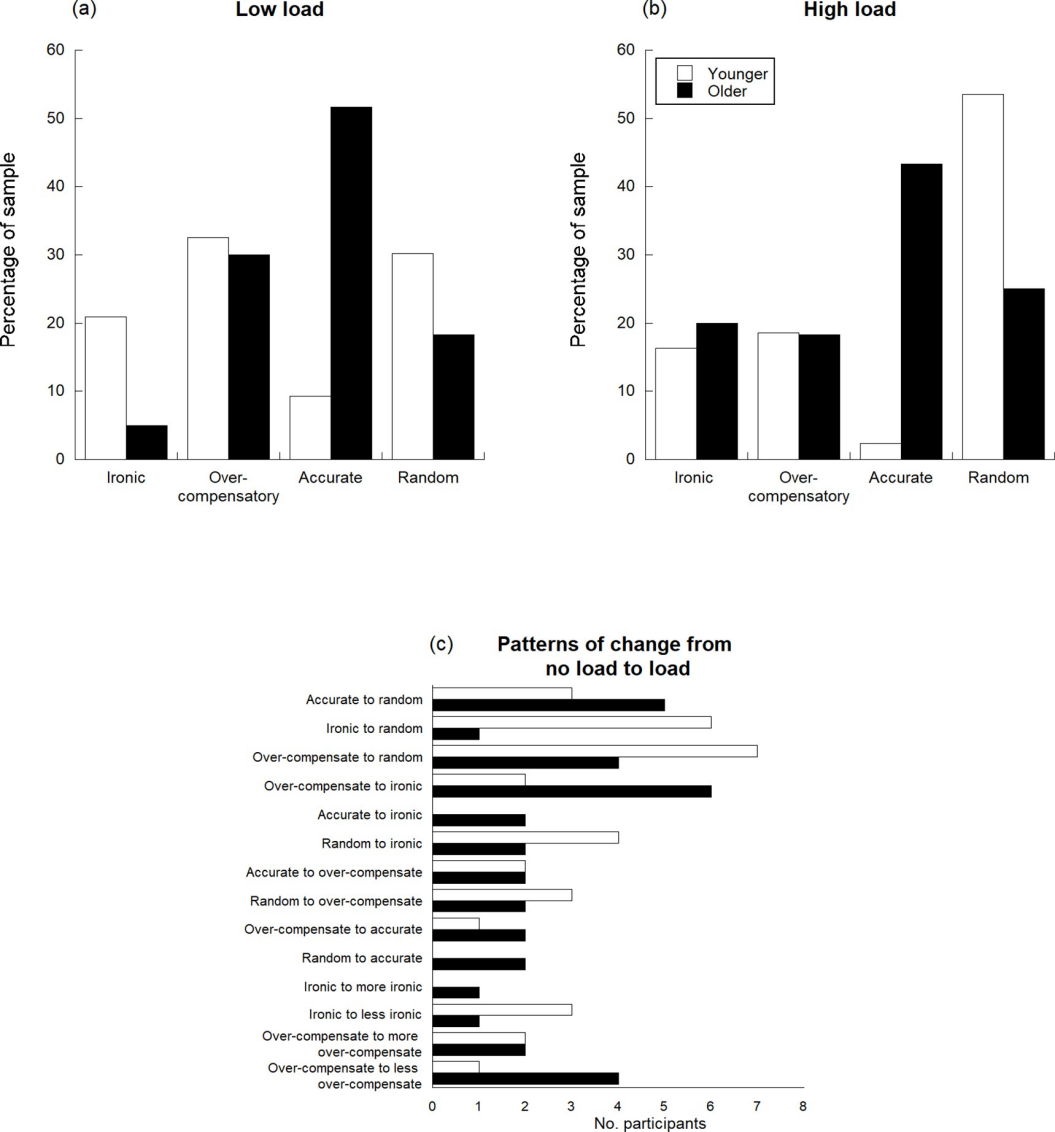
(a) the percentage of participants in each response category under low load, (b) under high load, and (c) patterns of change from low to higher load. N = 39 for the younger sample and N = 64 for the older sample.

To test for age differences in ironic errors independent t-tests were conducted on both the percentage and magnitude of errors (Tables 1 and 2). This showed that older adults made significant fewer ironic errors than the younger adults (t(101) = 5.15, p < .001) and the magnitude of their ironic errors was smaller (t(101) = 3.42, p = .001). Fig 2 also shows that under the no load condition only 5% of the older adults were categorised as ironic performers compared to 21% of the younger adults. These findings do not support the hypothesis of an age-related increase in susceptibility to making ironic errors.

**Table 1:** Means and SDs for the percentage of movements in the avoidant instruction conditions that were accurate or demonstrated ironic or over-compensatory errors, across both age groups and memory load conditions.

		Ironic		Over-compensatory		Accurate	
		Mean	SD	Mean	SD	Mean	SD
Younger	Low load	34.13	13.93	38.90	15.66	26.97	15.68
	High load	37.10	11.61	36.26	13.76	26.64	11.19
Older	Low load	18.52	15.44	36.67	21.30	44.81	25.96
	High load	25.49	19.90	28.91	17.98	45.60	25.20
Totals							
Low load (averaged across age)		26.33	14.69	37.78	18.48	35.89	20.82
High load (averaged across age)		31.30	15.76	32.58	15.87	36.12	18.20
Younger (averaged across load)		35.62	12.77	37.57	14.71	26.81	13.44
Older (averaged across load)		22.01	17.67	32.79	19.64	45.20	25.58
Overall (averaged across age and load)		28.81	15.22	35.18	17.18	36.01	19.51

**Table 2:** Means and SDs for the amplitude (cm) of ironic and over-compensatory errors across both age groups and memory load conditions.

		Low load		High load	
		Mean	SD	Mean	SD
Younger (n = 39)	Ironic deviation (cm)	13.74	7.73	16.00	8.61
	Over-compensatory deviation (cm)	21.15	14.23	15.51	10.22
Older (n = 64)	Ironic deviation (cm)	9.25	5.58	13.23	10.11
	Over-compensatory deviation (cm)	20.75	16.81	13.77	9.99
				Mean	SD
Totals (n = 103)	Low load (averaged across error deviations and age)			16.22	11.09
	High load (averaged across error deviations and age)			14.63	9.73
	Younger (averaged across error deviations and load)			16.60	10.20
	Older (averaged across error deviations and load)			14.25	10.62

To establish whether older adults over-compensated more than younger adults independent t-test were conducted on the percentage and magnitude of over-compensatory errors. These showed no significant age differences (percentage; t(101) = .57, p = .57, magnitude; (t(101) = .125, p = .901). Overall 33% of the younger and 30% of the older adults were categorised as being over-compensators under no load. These findings did not support the notion of the older adults adopting a ‘play safe’ strategy either.

The next set of analysis examined the impact of additional cognitive load. In line with the Ironic Processing Model it was predicted that ironic errors would increase under higher load and this would be greater for older than younger adults. It was also expected that older adults who were classified as ironic performers under low load would also be ironic performers under high load. We conducted a two-way ANOVA (age (younger, older) x cognitive load (low, high)) on the percentage of ironic errors. The main effect of cognitive load was significant (*F*(1,101) = 9.65, *p* = .002, η_p_^2^ = .09) with more ironic errors being made under cognitive load, but the interaction between load and age was not significant (*F*(1,101) = 1.53, *p* = .22, η_p_^2^ = .02). The main effect of age was significant (*F*(1,101) = 22.60, *p* < .001, η_p_^2^ = .18) as described above.

Similarly, a two-way ANOVA (age (younger, older) x cognitive load (low, high)) on the magnitude of ironic errors showed a main effect of load (*F*(1,101) = 9.42, *p* < .01, η_p_^2^ = .09) with an increase in magnitude under load. The interaction between age and load was not significant though (*F*(1,101) = .72, *p* = .40, η_p_^2^ = .01). The main effect of age confirmed older adults made smaller errors (*F*(1,101) = 7.64, *p* < .01, η_p_^2^ = .07). Only three younger (7%) and two older (3%) adults were consistent ironic performers and in all but one case the magnitude of ironic errors increased under load. Together these findings do not provide strong support for ironic action errors being a dominant behaviour, nor do they support the prediction that older adults would be more susceptible than younger adults to ironic monitoring under cognitive load.

To assess whether there were age increases in implicit compensation, a significant age x load interaction where older adults would have greater over-compensatory errors under load compared to younger adults was predicted. A two-way ANOVA (age (younger, older) x cognitive load (low, high)) on the percentage of over-compensatory errors found the main effect of cognitive load to be significant (*F*(1,101) = 6.86, *p* = .01, η_p_^2^ = .06), but less rather than more over-compensatory errors were made under cognitive load. The interaction between load and age was not significant (*F*(1,101) = 1.66, *p* = .20, η_p_^2^ = .02). The same pattern was found for the magnitude of over-compensatory errors; main effect of load (*F*(1,101) = 13.31, *p* < .01, η_p_^2^ = .12) with decreases under load and a non-significant interaction (*F*(1,101) = .15, *p* = .70, η_p_^2^ < .01). Only 7% of the younger and 9% of the older adults were classified over-compensators under both conditions.

Individual differences in performance were noted within both age groups, with some individuals showing a predominantly consistent pattern of responding across the cognitive load conditions whilst others were inconsistent. Fig 2, showing the percentage of younger and older adults in each response category, highlights the higher levels of accuracy shown by older adults. Only 5% of the older sample consistently made ironic errors under the low cognitive load condition, compared to 20.93% of the younger sample. Under low load an age by performance category chi-square analysis showed a significant difference between expected and observed frequencies of performance category for the two age groups (χ^2^(3) = 20.25, *p* < .001). As illustrated in Fig 2A fewer older adults were classified as over-compensators, ironic or random performers compared to the younger adults, and the number of older adults who were accurate performers was greater than younger adults.

Fewer older (44%) compared to younger (70%) adults changed their dominant response style under cognitive load, and the age by performance category chi-square test under cognitive load revealed significant differences in these distributions (χ^2^(3) = 20.33, *p* < .001). As shown in Fig 2B the number of older and younger adults who were classified as ironic or over-compensatory performers was similar, but more older adults were accurate and fewer performed randomly compared to younger adults. Comparing across the load conditions, the number of older adults who made ironic errors increased from the low to high load conditions as expected, but the number of people in both age groups who made over-compensatory errors reduced.

#### The Simon task

As predicted, older adults were significantly slower on congruent trials (*t*(101) = 2.58, *p* = .011) and their inhibition times (RT incongruent–RT congruent) were significantly longer than the younger adults (*t*(101) = 8.10, *p* < .001). Inhibition times as a proportion of response times on congruent trials were also compared, revealing a significant difference between age groups (*t*(101) = 24.44, *p* < .001) with older adults showing a higher proportion (mean = .58) than younger adults (mean = .31).

#### Relationships between the avoidant instruction errors and the Simon task

To determine if the ability to inhibit avoidant instructions and the ability to inhibit a habitual motor response were related Pearson’s correlations were conducted on the percentage of ironic errors under each load condition and the proportional inhibition scores on the Simon task. Proportional scores were used to account for age-related slowing. Under no load there was a significant negative correlation (r = -.45, p < .001) and this reduced slightly under load (r = -.32, p < .001). The other error measures on the avoidant instructions tasks were also correlated with the proportional inhibition scores and no significant relationships were found. To check whether age-related slowing was related to error tendencies mean response time on the congruent trials was correlated with the percentage and magnitude of both error types under both load conditions. No significant correlations were found.

## Discussion

Similar to previous studies [30–34] older participants showed significantly longer inhibition times than younger participants on the Simon task even after accounting for age-related slowing. Older adults thus showed age-related decrements in both processing speed and the ability to inhibit a natural perception-action response. Unexpectedly however, older adults outperformed younger adults on the avoidant instruction task; when instructed not to move in a certain direction, older adults made significantly fewer ironic errors and the magnitude of their errors was also smaller than younger adults. There were no significant age differences in the percentage or magnitude of over-compensatory errors indicating that overall the older adults were surprisingly better at coping with the error-provoking instructions compared to younger adults.

We had predicted that we would see age-related declines in either ironic monitoring or implicit over-compensation. The findings did not support either of these two predictions. We also predicted that when given an additional cognitive task older adults would either show a greater increase in ironic or over-compensatory errors than younger adults, as the additional cognitive demands placed extra pressure on the resources available for controlling actions, or they would make fewer errors than younger adults if the secondary task acted as a distraction from the avoidant instructions. Again the results did not fully support these predictions is as both age groups demonstrated similar increases in ironic errors and decreases in over-compensatory errors under cognitive load. However, the presence of some ironic behaviours was consistent with the Ironic Processing Model [20]. That is when told *not* to do something, attempts to inhibit the prohibited action, ironically caused the monitoring system to assume control of action and thus prompt those ironic actions to occur. This model however does not account for the greater prevalence of over-compensatory compared to ironic errors across both age groups, although this is explained by the implicit compensation hypothesis [45] that over-compensatory errors are caused by negatively worded instructions exaggerating the importance of the movement to be avoided, leading to the misconception that it is less risky to err away from the forbidden direction rather than towards it. Thus, under avoidant instruction this ‘play safe’ over-compensation strategy seemed to be the default process for 38% of the total sample as oppose to 26% of the sample who made ironic errors.

We also examined the extent to which ironic errors during the avoidant task correlated with proportional inhibition time on the Simon task. It was reasoned that if they shared a common inhibitory process then a significant positive correlation would be found. Our findings of significant negative correlations for both load conditions directly opposed the predicted direction of results. This suggests that there is not a single common process underlying these two behaviours but when faced with avoidant instructions, older people may employ different strategies or undergo different processes than younger people which allows them to perform better, although it is not possible to tell from our data which strategies or processes are involved.

Why older participants outperformed younger adults however is not clear. This was not caused by age differences in speed-accuracy trade-offs [47], as there were no significant differences between age groups in movement durations, amplitudes, peak velocities and end-point positions, thus older people were not slower or less accurate than younger adults. Moreover, mean response times for the congruent trials on the Simon task were not significantly correlated with any of the avoidant instruction error measures indicating that the more error prone participants were not slower. It is also unlikely that the older adults’ superior performance was caused by not engaging in rehearsing the 7-digit number, as their recall was no poorer than the younger adults. Indeed the lack of age decrements in both digit recall and motor errors suggests that older adults did not need to prioritize motor control over cognitive performance to perform well, in contrast to findings from other cognitively demanding action tasks such dual walking-while-talking [48].

An examination of the literature points to two potential frameworks of normal age-related changes that could be explored as potential explanations for our findings; greater proactive emotional regulation, and increased vulnerability to distraction. Research shows that older people use more proactive emotion regulation strategies as an adaptive strategy to prevent negative affect (and maintain positive affect) in the face of age-related declines [49]. Such strategies are thought to be worth the cognitive effort, as the negative affect resulting from poor performance is too costly to manage in the long term. Applied to our own study, this could suggest that the reduced prevalence of ironic errors was influenced by an age-related cognitive strategy whereby older adults employed enhanced effort to perform well and thus maintain positive affect, which may have succeeded for the avoidant instruction task but not the more effortful Simon task. However, if the older adults were employing such a cognitive strategy then the expectation would be that they would show a greater performance decrement under cognitive load than the younger adults, but this was not the case as both groups showed similar increases in ironic errors and decreases in over-compensatory errors. It may have been the case however that the digit recall task was not sufficiently complex to detrimentally affect the performance of healthy older participants. For example age decrements under cognitive load may be more pronounced when the secondary cognitive task involves e.g., spatial working memory or backward digit recall [50]. Thus whether and how older adults employ enhanced effort to maximise performance under avoidant instruction requires further research.

The superior performance of older adults overall, might be explained however by the argument that normal ageing also increases vulnerability to distraction which can inadvertently benefit cognitive performance [39]. For example, applying this to our avoidant instruction task, age-related increases in distractibility may have distracted older people’s cognitive resources away from the error-provoking instructions, and this may have been aided by the introduction of the secondary cognitive task. Such distraction from the avoidant instructions may have inadvertently increased the cognitive resources available to focus on producing accurate movements, thus resulting in fewer ironic errors under low load and a reduction in the more dominant over-compensatory error type with load. Whilst there was partial evidence to support this in that older adults made fewer ironic errors under no load and over-compensatory errors decreased with load, there was an increase in ironic errors under load and there was no significant interaction effect (load x age) for either the percentage or magnitude of ironic or over-compensatory errors. However, seven participants changed their error pattern from ironic to random, eleven from over-compensatory to random and three from over-compensatory to accurate (20% of the total sample) under load indicating that the addition of the cognitive load reduced error rates for some participants.

In summary thus far, the Ironic Processing Model [20] may account for the occurrence and magnitude of ironic action errors under avoidant instruction, and the implicit compensation model [45] may account for the greater occurrence of over-compensatory errors, although older adults seem to be less prone to both sets of processes than younger adults. Instead, the unexpected superior performance of older adults suggests that they may engage in different processes to younger adults when under avoidant instruction, although the nature of these remain unknown. It is unlikely that the older adults were more driven by emotion-regulating strategies (e.g., enhanced effort), but it is possible that age-related increases in distractibility helped the older adults more than younger adults when dealing with avoidant demands. A further speculation that may account for our findings is that during the early phase of motor skill learning making errors is an essential part of the process and critical to refining and developing a generalised motor programme. It may be therefore, that younger adults who are still actively acquiring new motor skills are less reticent about making mistakes during the learning process than older adults who actively have to monitor and control their actions to avoid falls, slips or bumps.

Individual differences in error tendencies were also found in both older and younger adults. Some participants made predominantly ironic errors, whilst others were over-compensators. In addition, a similar pattern of individual differences was seen in the changing error types produced under cognitive load with more younger (70%) than older adults (44.4%) performing differently under the two load conditions. Compared to the no load condition, older adults were more prone to making ironic errors under cognitive load as predicted (an increase from 5% to 20%), and the number who performed accurately reduced (51.67% to 43.33%). This supports the model of ironic processing, although the reduction in the number of older people making over-compensatory errors (30% to 18.33%) was not expected, although is in line with the suggestion that distraction from the error-provoking instructions may have aided performance. Older adults changed from being over-compensators to ironic performers (n = 6), to being more accurate (n = 2) and to showing a random pattern of responding (n = 4), indicating that a variety of factors are likely to drive these behaviours. It is not clear however why different individuals may be more prone to different error types. One recent study shows that susceptibility to ironic behaviours is greater amongst those with neurotic personality traits [51] which makes sense given that thoughts linked to neuroticism (e.g., worrying about evaluation, failure, and aversive consequences) can load working memory, divert resources from the task in hand, and thus detrimentally impact performance [52]. The individual differences that predict susceptibility to ironic and other motor errors with older age requires further investigation, and other personality traits such as conscientiousness and openness needing to be explored.

Considering performance across both inhibition tasks, the presence of age-related decrements on the Simon task but age-related performance improvements on the avoidant instruction task suggests that the ability to inhibit a prepotent perception-action coupling and the ability to inhibit a prohibited action involve independent processes in older age which likely do not share the same neural circuitry. This is in line with evidence from brain imaging studies showing that age effects in patterns of brain activity differ between inhibition tasks. For example, in an fMRI study with participants ranging in age from 20–77 years [53], it was found that with increasing age, activity declined in the right inferior frontal cortex (IFC) during a stop-signal task (inhibiting an already planned action), but increased in the fronto-parietal regions during go/no-go (inhibiting interference between competing responses) and Simon (inhibiting the production of a pre-cued action) tasks. Further research is needed to ascertain whether these different patterns of brain activation between younger and older age groups are the result of selective age-related degeneration in the neural networks underlying some abilities, or an effect of implicit or explicit auxiliary strategies employed by older people to compensate for age-related declines. The neural mechanisms underlying the processing of avoidant instructions remain unknown, however as older adults engage in different processes than younger adults then we would expect them to show different patterns of brain activation on this task.

Applying our study to everyday contexts, our findings are relevant to how older and younger adults may cope with everyday avoidant instructions and how they learn new skills. For example, instructions such as ‘do not cross the line’, ‘do not press the red button’, ‘do not touch' could all induce ironic errors and our findings suggest that younger adults would be more error prone than older adults. Similarly, when learning a new skill giving younger adults instructions to focus on the action they wish to perform rather than the action they wish to avoid (e.g., "leave the starting block on time" rather than "don't leave the starting block early") is likely to be beneficial whilst older adults are less likely to be disadvantaged by avoidant instructions. Our evidence suggests that it is the younger, rather than the older, that may need the type of instruction adjustment, although future research is needed to test this.

In conclusion, this is the first study to investigate age effects in the tendency to make ironic action errors under avoidant instructions. Surprisingly, older adults who performed more poorly than younger adults on the Simon task outperformed younger adults on the avoidant instruction task. Our findings are limited by fewer younger than older adults being included due to incomplete data sets, thus our younger sample might under-represent the abilities of younger adults in general. Similarly, our older sample might under-represent age decrements in the general older population. Additionally, we did not record individual differences such as demographic, dispositional, or lifestyle variables, so we were unable to assess whether these are associated with our unexpected findings. As neuroticism has been shown to be related to the prevalence of ironic errors [51] it would be of value to explore whether this or other variables impact of the age-related advantage we found. The nature of the task we used to explore ironic errors also needs to be considered; in our task there were only two ways to make an error and it would be interesting to examine tasks where there are many action options available, only some of which will results in errors. Future research is needed to replicate our findings, investigate whether older people employ particular strategies (such as enhanced effort via emotional regulation strategies) or are benefitted by the consequences of other normal age-related cognitive changes (such as increased distractibility), and determine the extent to which individual differences affect susceptibility to making ironic and other action errors in older age.
